# Overcoming treatment gaps in the management of depression with non-pharmacological adjunctive strategies

**DOI:** 10.3389/fpsyt.2023.1268194

**Published:** 2023-11-28

**Authors:** György Purebl, Katharina Schnitzspahn, Éva Zsák

**Affiliations:** ^1^Institute of Behavioral Sciences, Semmelweis University, Budapest, Hungary; ^2^European Alliance Against Depression, Leipzig, Germany

**Keywords:** depression, unmet need, treatment gap, prevention, pharmacological treatment, psychotherapy, synergism

## Abstract

There is considerable evidence that simple, cost-effective, non-pharmaceutical strategies can be readily implemented to improve outcomes in the treatment of depression. It is estimated that 4.4% of the world’s population suffers from depression. Despite being a major public health concern and the availability of both pharmacological and non-pharmacological treatments, many depressed people remain undiagnosed and receive no or inappropriate treatment. Several possible underlying factor of treatment gap can be identified in relation to pharmacotherapy and psychotherapy of depression, including side effects, partial remission, treatment-resistant depression and the limited availability of psychotherapy. In addition to developing new therapeutic options, much more could be done to optimise the use of existing therapies, including combining available drug treatments with quick, simple and cost-effective non-pharmacological methods: low-intensity psychological interventions, online self-help tools and lifestyle medicine. In addition to increasing the effectiveness of treatments, prevention is equally important: awareness programs to further reduce the treatment gap, and community dissemination of the life skills that help maintain positive mental health.

## Introduction

It is estimated that 4.4% of the world’s population suffers from depression (1). Its prevalence has increased by 54.3% between 1990 and 2013 (2), and in spite of many efforts and millions of euros of investment worldwide, unipolar depression has become the leading cause of disability (3). Despite being a major public health concern and the availability of both pharmacological and non-pharmacological treatments, many depressed people remain undiagnosed and receive no or inappropriate treatment (4–6). Several unmet needs can be identified behind these grim figures.

## The treatment gap of depression treatment

There is a “negative cascade” in the treatment of depression (7), meaning that although there are many patients with depression, few reach appropriate care, fewer receive appropriate treatment, and even fewer are adherent to treatment—so that only a small fragment of patients benefit from effective treatment. Although socio-economic factors also contribute (8, 9), significant proportion of patients do not seek help for their symptoms on their own, for couple of reasons. Firstly, they feel they are beyond help—hopelessness is a symptom of depression (10). Secondly, stigma and negative beliefs towards mental health services also contribute in the low frequency of help-seeking behavior in depression (11, 12). Thirdly, depressive experience can be widely misperceived (13), sometimes underestimated: some believes that their symptoms are natural feelings resulting from a negative life event. Those who do consult a primary care physician do not usually wish to see a psychiatrist or other kind of mental health professional initially (11), and they reports mainly somatic complaints (14), and the psychological symptoms are less intensive (in the prodromal phase) or kept hidden (15). This presentation of symptoms can be misleading—the depression remains undiagnosed and inappropriate treatment is often used: sleeping pills are prescribed for sleep problems, vitamins are recommended for fatigue, and the somatic symptoms may prompt expensive and unnecessary diagnostic procedures, while the underlying depression is not resolved. Ultimately, only a minority of patients with depression receive appropriate treatment (16). According to the WHO’s World Mental Health Survey, only 56.7% of participants who met the diagnostic criteria for depression reported needing treatment. Of those who recognized their need for treatment, only 71.1% made at least one visit to a health care facility. Of those who finally received treatment, only 41.0% received the minimum standard of care, and only 16.5% of all people diagnosed with depression received minimally adequate treatment (4).

However, antidepressant treatment alone is no guarantee that the patient will receive adequate treatment: under-dosing (17) or low response rates to the first antidepressant regimen (18) are common, which can undermine the fragile optimism and motivation of clients who, by the nature of their condition, are often characterized by hopelessness. Even where treatment is adequate, adherence problems are common (19, 20), with a non-adherence rate of approximately 50% (21)—which may lead to the underestimation of the benefits of antidepressants (22). Treatment may be stopped at early signs of remission (the client thinks there is no need to further treatment) or because of a side effect. In many cases, either being treated with medication or psychotherapy, the reduction of the symptoms, does not bring the change that clients really expect. Full functional recovery and quality of life improvement takes longer than symptom remission (23).

With these factors in mind it appears that most current treatment options, although clinically effective, have significant limitations and often fail to meet patient’s needs or expectations.

## Unmet needs about the pharmacological treatment of depression

As with any long-term treatment, it is important for antidepressants to minimize side effects that reduce quality of life, thereby potentially compromising long-term patient compliance. Although there is considerable individual variability in patient response to the detectably different antidepressant molecules (18), the most important side effects of antidepressants according to a recent large-scale longitudinal study has documented that the most problematic antidepressant side effects are weight gain, sexual dysfunction and sleep problems (24). However, achieving higher level of compliance and adherence is not only a matter of side effects: other important factors also include socio-economic status, co-morbidities, physician attitude and communication about side effects. Nevertheless, a better side-effect profile is an important need for both physicians and patients.

Conversely, side effects might be more tolerable if the available antidepressants were more effective at reducing the distressing symptoms of depression. An example is the slow onset of action, common to all conventional antidepressants in varying degrees. Patient adherence and satisfaction would almost certainly improve if antidepressant agents were able to reduce symptoms much more rapidly, spectacularly, and reliably. As it is, up to 30% of patients do not respond satisfactorily to conventional treatment, and designated as treatment-resistant (25).

Another vexing problem is that there is no reliable method for predicting which antidepressant will prove effective for a patient, with the result that many patients do not respond to the first choice of medication (18, 26). Moreover, evidence remains inconsistent on the optimal time to wait before switching medications (26, 27). Even after identifying an effective antidepressant agent, an additional problem is residual symptoms that impair quality of life, when a patient terminates treatment after partial remission (28–30). The most common of these are residual sleep disturbances, somatic complaints, fatigue, impaired concentration (29, 30). All of this makes it difficult to return to work and daily life in general, and also increases the risk of relapse.

Despite the broad repertoire of current medicines that work well for a significant proportion of patients, there persists an unacceptably high rate at which these treatment options fall short of effecting full recovery and restoration of quality of life.

## Treatment gap in the non-pharmacological treatment of depression

A significant proportion of depressed patients can be treated with evidence-based psychotherapy (31–33). However, access to psychotherapies varies considerably between countries. In some countries, psychotherapy care is well institutionalized and adequately funded; in others, patients face long waiting lists for therapy; and even today, there are countries in Europe where psychotherapy is mostly available only in private practice (34). Cultural stereotypes can also hinder acceptance of psychotherapy—patients simply do not use it, even when other treatment options do not work. Thus, limited access, funding problems and cultural stereotypes result that the potential of evidence-based psychotherapies is not yet being adequately exploited in the treatment of depression.

Bright light therapy (BLT), first mentioned by Aretaeus of Cappadocia in the second century A.D. with its robust chronobiological effect (35), is probably the oldest method for the effective treatment of mood symptoms. Although originally considered a first-line treatment for seasonal affective disorder (SAD), increasing evidence supports its efficacy also in non-seasonal depression (35–37), either as monotherapy (36) or to augment pharmacotherapy (37). Despite its efficacy, the potential of BLT is not fully exploited in daily clinical practice, and the procedure still needs to be standardized in terms of exposure intensity and treatment duration (36).

The sharp rise in the prevalence of depression suggests that lifestyle may play a role in the development of depression. Large body of convincing scientific evidence links risk of depression to sleep deprivation, disrupted circadian rhythms and sedentary lifestyles (38–41). Conversely, sleep management, a healthy diet rich in fish, fruit and vegetables, and regular exercise have strong potential to reduce the risk of depression (39, 40). These factors are also strongly interrelated: regular exercise has important rhythm-entrailing effects, promotes sleep, and is effective in reducing stress. There is considerable evidence that components of a healthy lifestyle produce epigenetic changes similar to antidepressant medication, for example in the BDNF systems (41).

The potential of lifestyle medicine in depression is therefore very promising, but has serious limitations in everyday practice. Achieving lifestyle change is at least as difficult in depressed patients (particularly during the depressive episode) as it is in other major public health concerns.

## Overcoming treatment gap I. Advances in the pharmacological treatment of depression

Several recommendations are available for managing partial remission and treatment-resistant depression, from augmentation strategies to optimizing, switching and combining the available antidepressants (42, 43). In addition to pharmacotherapy, various brain stimulation techniques are being developed, ranging from transcranial magnetic stimulation to deep brain stimulation (42, 44).

Pharmacological research also continues to address unmet needs, and new molecules are expected to emerge in pharmacotherapy. Some of these continue the search for fewer side effects and more complete clinical improvement, and the desired goal (for patients, physicians and manufacturers alike) is a compound with rapid onset of action, full recovery potential and minimal side effects. However, a new paradigm has emerged that shifts the focus from symptom reduction to the restoration of a rich emotional life, the remission of cognitive symptoms, a significant change in quality of life and the ability to regain productivity and work capacity as quickly as possible. There are a number of novel therapeutic agents that may have a transformative effect on the day-to-day management of depression (45).

The first and probably the most intensively studied representative is the novel line is ketamine (more precisely its enantiomer, esketamine), a rapid acting NMDA antagonist compound, with convincing antidepressant properties (46–48). So far, esketamine has FDA and EMA approval for the therapy of treatment-resistant depression, and future studies will explore its full potential in the treatment of mood disorders (and perhaps other psychiatric conditions). The next promising object of research is psilocybin, a partial 5HT receptor agonist with strong psychoactive potential (42).

Since an inflammatory model is also hypothesized as a possible contributor to depression symptomatology, potent anti-inflammatory compounds are also knocking on the door of depression medicine: infliximab (a TNF-alpha antibody widely used in inflammatory diseases from rheumatoid arthritis to inflammatory bowel disease) or brexanolole, a progesterone derived compound with neurosteroid properties (approved for the treatment of postpartum depression). In sum, the landscape of pharmacological treatment for depression is set to change significantly in the near future.

It is important to note that although the pursuit of more advanced compounds and the commitment to broadening the treatment repertoire for depression are very important, the available alternatives are often not fully exploited in daily practice. Outcomes can be further improved by combining antidepressants, augmenting them and adding non-pharmacological treatment options: lifestyle changes, self-help programs, psychological interventions and psychotherapies. Even simple interventions (e.g., behavioral activation, use of decision aids, shared decision making, motivational interviewing) or online self-help tools can improve the matching of therapy to patient preferences and increase the effectiveness of antidepressant treatment (49–51).

## Overcoming treatment gap II. Advances in non-pharmacological treatment

In psychotherapy, there is a pressing need for further research to determine which therapeutic methods are effective in depression (31–33). Wider access to evidence-based psychotherapies, too, would certainly be welcome—psychotherapy should not be the privilege of a narrow social group. The emergence of internet-based psychotherapies promise a major advance in this respect. These are easier to combine with pharmacological treatment than face-to-face therapies, as they require much less personal presence. With appropriate motivation, they can be as effective as in-person therapies, but are much more accessible and cost-effective (52–57).

Online guided self-help interventions, such as the iFightDepression^®^ tool (49, 52, 53) from the European Alliance Against Depression (EAAD) can also be a highly accessible and effective treatment addition. The tool uses principles of cognitive behavioral therapy to support self-management of depressive symptoms in individuals with milder forms of depression. The effectiveness of the iFightDepression^®^ tool in reducing depressive symptoms has been demonstrated by a randomized controlled trial with an active control group (50) and another study with a treatment as usual control group (49).

Online self-help tools are not the only simple but effective psychological interventions that can be recommended to clients. There are low-intensity evidence-based in-person psychological interventions that take little time and can easily be delivered by non-specialist healthcare providers. The IAPT (Increase Access to Psychological Therapies) program aims to expand psychotherapy services in the United Kingdom with a stepped care model that starts with single psychoeducational interventions and progresses to specialized psychotherapies. IAPT has developed, implemented and demonstrated the effectiveness of a range of low-intensity psychological interventions, including for more severe forms of depression (58, 59). As a result of its success, IAPT has become a model for the development of similar services in other countries. To make psychological support more accessible in low-and middle-income countries, WHO has also developed a set of simple psychological interventions that can be learned not only by health professionals but also by lay people (60, 61). The use of these tools by non-specialists (e.g., social workers, school teachers, priests or simply motivated lay helpers) can further increase access to psychological help and may serve as a quick first response with considerable preventive potential for more serious mental health problems. In summary, there are many simple psychological interventions that can be learned by any health care professional—or by engaged lay helpers. These are easy to learn, take little time to implement, but are evidence-based, and can significantly increase the professional’s personal effectiveness in successfully treating depressed patients. In addition, wider use of these simple but effective tools may help to identify strategies to improve overall wellbeing and coping with life adversities. These strategies may form the basis of cost-effective community mental health promotion programs.

Another important goal is to integrate lifestyle counselling into psychiatric practice (62, 63), as it has already been done in cardiology and diabetology. Although it may be more difficult to motivate clients with mental health conditions to change their daily diet and sedentary lifestyle, the psychological interventions mentioned above may help to improve cooperation from clients.

## Overcoming treatment gap III. Tackling depression at the community level

The “depression pandemic” demands much more from health care than advances in psychiatry. Depression should be recognized in areas of medicine other than psychiatry and primary care. Depressed clients can present in all types of health care settings due to the high prevalence and frequent comorbidities of depression (64–66).

We may go further; responsibility of tackling depression also goes beyond healthcare issue and concerns society as a whole. Knowledge of depression must be communicated to the public to dispel misconceptions, combat stigma and discrimination, and promote lifestyle factors that reduce the risk of depression in general. It is equally important to provide lay people with practical skills to recognise symptoms, support people with depression, facilitate help-seeking behavior and, most importantly, to establish and maintain their own positive mental health. Several awareness campaigns have been carried out in the past to increase knowledge about depression, but the current situation, low treatment rates, stigma and misconceptions associated with depression suggest that something different and more effective is needed.

In addition to public campaigns, there are a number of other (and probably more effective) approaches to increasing knowledge about depression in the community. School-based programs can be effective in providing recognition and support skills training for both teachers and students, as demonstrated for example in the European SEYLE project (67).

Multilevel and multifaceted interventions are another approach. The community-based 4-level intervention from the EAAD which simultaneously addresses depression and suicidality, has been identified as the most promising multifaceted and evidence-based intervention by a recent systematic review (68). It combines evidence-based prevention, education and treatment methods that are tailored to the needs and resources of local communities (53, 69). EAAD’s 4-level approach involves primary care physicians and other primary care health professionals (Level 1), local media and the general public (Level 2), community facilitators (such as social workers, priests, pharmacists, journalists, teachers) and key stakeholders (Level 3) and patients and their relatives (Level 4). It has been successfully adapted to different cultures and health care systems both within and outside of Europe. Being simultaneously active at four intervention levels, it can maximize the synergy between different types of interventions (70). Low-threshold services (such as telephone helplines) and the iFightDepression^®^ tool described above are integral parts of the approach.

The work environment, where people spend much of their time, is a significant source of daily stress and challenges (71, 72). Workplace-based mental health promotion therefore has a large and under-exploited potential for tackling depression. There have been some effective programs for addressing depression-related issues at work (73–76), but workplace mental health programs are not available to the majority of workers, especially in small and medium enterprises. Reviewing and evaluating the experiences of previous initiatives, the EU-based MENTUPP program offers a multi-level approach in three sectors (healthcare, construction, ICT), targeting both clinical and non-clinical manifestations of depression, anxiety and stress-related problems, and promoting a non-stigmatizing, mutually supportive working environment. The MENTUPP Hub, an online platform presenting interactive psycho-educational materials, toolkits and links to additional resources, is a key element of the program (77). The preliminary results of MENTUPP demonstrate that workplace-based interventions have the potential to improve mental health and employees’ wellbeing (78).

To successfully tackling depression (and mental disorders in general) there is a clear need for more psychoeducational activities in the places where people spend most of their time and interact with each other—in schools, workplaces and other community settings. Rather than only focusing depression-related issues, these psychoeducational activities should also work the life skills of maintaining positive mental health.

## Unanswered questions and future dilemmas

Many people with depression are difficult to treat, despite the fact that there are numerous available and evidence-based treatments. This in itself is evidence that depression is a very heterogeneous condition, underlining the importance of personalized treatment. A major step forward would be the ability to predict which individual combination of therapeutic modalities will result in a rapid and complete recovery for each patient. This could be approached using a number of technologies that may be available in the near future, involving practical pharmacogenomics, more advanced knowledge of the inflammatory, hormonal, etc. processes involved in depression, the individual cognitive psychological characteristics. All of these could be usefully incorporated into a comprehensive measure for predicting therapeutic response. Other important questions are how the many approaches may be integrated into a cost-effective framework, and how, amid the simultaneous use of many therapeutic modalities, a balance may be found between highly personalized therapy and quality-controlled, guideline-driven treatment protocols.

There are some promising initiatives for integrating the different approaches into multi-modal treatment regimes. Many of these approaches can be channeled into a comprehensive framework via the concept of a transformative treatment model for mood disorders (79). Transformative models involve trait and state symptom characteristics, possible staging models, predisposing elements and possible future trajectories of the course of depression (80–82). Treatment response in this approach, goes beyond reduction and functional recovery to take account of exacerbations, relapses and other specific indicators such as hospital admissions, number of psychotropic medications and self-harm behavior (79–82). Evaluation of the multimodal approach and incorporation of predictive measures may also be feasible in naturalistic studies (82, 83) and multifaceted community interventions (68–70).

Prevention is an area that demands at least as much emphasis as therapy, if not more. Much promising research has already been published, providing (albeit limited) evidence of the potential beneficial effects of various lifestyle factors. There are several questions need to be answered. What are the key life skills and positive psychology concepts (e.g., savoring and creative and executive efficiency) that help to maintain positive mental health (84)? How can these skills be transferred and scaled up to community level? What are the ecological aspects of depression (and mental disorders in general) the effect of a person’s disconnection from nature, and how can the natural environment and access to green spaces contribute to prevention and treatment (85–87)? Recent reviews focus on how spirituality, a fundamental human dimension, relates to the pathology of depression and how spirituality may or may not be protective (88, 89)? A number of exciting research projects are emerging on these topics, and the number of models which possibly contribute to depression (and its prevention) is increasing. What all these new models have in common is that they are complex and emphasize the profiling of multiple factors (68, 70, 79, 83, 84, 87). Approaches that focus on the role of a single factor (e.g., a particular substance or a single psychological model) are likely to be less successful in the future understanding of depression.

## Limitations

This paper aims to review the progress and future directions of possible responses to the treatment gap of depression, but is not a systematic review. The references are mainly based on recent work, include systematic reviews, and there may be relevant studies that may not be mentioned here. For these reasons, the paper does, not have definitive recommendations on the response to treatment gap. Nonetheless, it should help to formulate more precise questions for the research and development of treatments of depression in the near future.

Depression—and mental disorders in general—is a growing public health concern with significant socio-economic implications, and we have attempted to give an overview of the many parallel efforts to prevent and treat depression. Instead of running in parallel, these would be much more effective if they work together taking full advantage of the synergies.

The increasing number of mental health problems demand more than targeted mental health promotion programs and the expansion of available mental health services. Mental health can also be improved through activities in other sectors, including social care, child and family welfare and legislation. These sectors are particularly well-placed to provide early interventions, especially in in high-risk groups, and rapid treatment for those already suffering from mental disorders. Prevention is crucial, but barriers to treatment for those already struggling with severe mental disorders must also be removed. Available mental health services must therefore be scaled up and mental health in general given higher priority, all of which naturally demands increased funding.

## Author contributions

GP: Writing – original draft. KS: Review and presentation of available literature about multi-level community-based program. EZ: Review and presentation of available literature about BLT and TMS and contribution of the elaboration of the final structure of the MS.
